# Students’ appraisal of a preparedness model for the provision of oral health care during a pandemic

**DOI:** 10.1186/s12903-022-02535-1

**Published:** 2022-11-16

**Authors:** Mario A. Brondani, Nasim Noroozbahari

**Affiliations:** 1 Department of Oral Health Sciences, Division of Dental Public Health, Faculty of Dentistry, University of British Columbia, 2199 Wesbrook Mall, Vancouver, BC V6T 1Z3 Canada; 2 Department of Oral Health Sciences, Faculty of Dentistry, University of British Columbia, 2199 Wesbrook Mall, Vancouver, BC V6T 1Z3 Canada

**Keywords:** Undergraduate dental education, COVID-19, Students, Preparedness, Model appraisal

## Abstract

**Background:**

The COVID-19 pandemic has impacted the provision of oral health care worldwide, prompting the discussion of preparedness. This study aimed to perform an initial appraisal of the usability, spatial representation, and clarity of a newly developed preparedness model from the perspective of senior undergraduate dental students at the University of British Columbia, Vancouver, Canada, enrolled in the 2020-21 academic year. Answers were analyzed thematically via an inductive coding process between March and June 2021.

**Results:**

All the 111 students in years 3 (#55) and 4 (#56) appraised the preparedness model, generating more than 200 pages of text. Four main themes were identified across the essays: streamlined depiction, information-based approach, adaptability to an ever-changing situation, and room for improvement. Although the majority of students appraised the model as being useful in fostering information-seeking behaviour, few students disagreed with the model’s portrayal and made further suggestions.

**Conclusions:**

Preparedness models can better guide oral health care providers during a health crisis such as a pandemic. The recently developed preparedness model was appraised as useful by senior undergraduate dental students, although alternative portrayals of the model were suggested. A comprehensive assessment of the newly developed model (and of its variations) is warranted to better support oral health care service delivery during a pandemic.

## Background

**Fig. 1:**
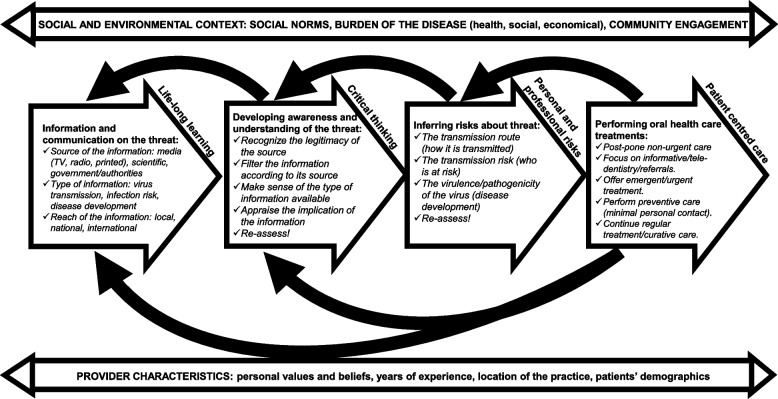
The preparedness model. Adapted from Brondani & Donnelly: A preparedness model for the provision of oral health care during unfolding threats: the case of the covid-19 pandemic. 6 BMC Oral Health 21, 254 (2021)

As of October 1 2022, the COVID-19 pandemic, caused by SARS-CoV-2, led to over 6.5 million deaths, and infected more than 625 million people worldwide [1]; new waves of infections caused by variants continue to stress the health care system of many countries. The COVID-19 pandemic has also negatively impacted dental education worldwide in terms of knowledge and clinical skills development [2–4]. The pandemic has also disturbed the provision of oral health care, particularly during the first wave of infections in 2020, due to the close proximity of professionals to patients’ mouths and noses—areas of easy transmissibility of this airborne virus [5]. Despite oral health care providers being prepared to mitigate the daily risks of pathogen transmission in their practices, the initial pandemic reopening plans put in place to resume oral health care likely happened as a reactive response [6] at least in Canada [7] likely due to the absence of a comprehensive preparedness model. Preparedness is understood as the knowledge and abilities to anticipate, respond to, and recover from the impacts of a health crisis such as the COVID-19 pandemic. Preparedness also requires an appraisal of the available (and ever-evolving) information about the pandemic, and of the communication plans and collaboration strategies to mitigate the crisis. One should also remain flexible and adaptive to changes in the disease progression and control of the spread of infections [8]. A recent study engaged 74 oral health care providers and their teams across British Columbia, Canada, to explore the barriers and facilitators to the provision of oral health care [6]. The information participants gave during that study led to the development of an empirical preparedness model for the provision of oral health care during an unfolding health crisis, using the COVID-19 pandemic as an example (Fig. 1). The model focuses on four areas: 1) life-long learning, 2) critical thinking, 3) personal and professional risk, and 4) patient-centred care.

The model shown in Fig. 1 emphasizes information and communication, development of awareness and understanding, inference of risks, and performance of oral health care during a health crisis or disease outbreak. The model also highlights the need to account for provider characteristics and skills set, and social and environmental contexts during a health crises, including the regulatory and licencing bodies and the health regulators [6]. But this model has yet to be appraised from different perspectives, including undergraduate dental students who may offer a unique input as future oral health care providers. Undergraduate students have also experienced the pandemic first-hand during their training over the past 2 years and may have specific understandings of proving oral health care in the midst of a health crisis [3]. The present study aimed to investigate the importance of dental students’ perspectives in appraising the newly developed preparedness model. Undergraduate third- and fourth-year senior dental students at the University of British Columbia’s Faculty of Dentistry were invited to freely discuss the model in Fig. 1 as we performed an initial appraisal of its usability, spatial representation, and clarity during their educational training in 2020-21. As future oral health care providers, it is important to gather students’ views on preparedness as they are about to enter the work force in the midst of an ongoing health crisis while being better prepared in the event of another outbreak.

## Methods

The methodology employed an exploratory qualitative inquiry that inductively and thematically analyzed the guided essays submitted by 55 DMD undergraduate third-year (27 males) and 56 DMD undergraduate fourth-year (29 females) students during the spring of 2021; as the essays were mandatory, the 111 students represented 100% of participation. The guided essays ranged from approximately 1500 to 2000 words each, in which students were asked to critically appraise the model shown in Fig. 1 in terms of its clarity, usefulness, comprehension, applicability, layout, and components. Students were also asked to highlight both positive and negative aspects of the model to avoid a socially desirable appraisal, or the idea that we were looking for a specific and more optimistic view of it. Students were instructed to also support or refute their arguments by citing up to five scientific references so they could explain how the model would enable them (or not) to be prepared for providing care during a health crisis. Students could also suggest changes in the components and/or layout of the model. As this essay was part of an undergraduate module in dental geriatrics, [9] it was submitted for formal grading between March 10 and June 1, 2021; this essay was worth 30% of the module’s final grade in year 3 and 42% in year 4, given the different grading scheme within each module. This was a take-home assignment where students had 2 weeks to complete; each essay was graded with an existing rubric according to the module’s syllabus. Although the topic of the COVID-19 pandemic (e.g., cause, disease development, societal impact, use of personal protective equipment) was taught in the geriatric module, none of the students received formal instruction or didactic content about preparedness in particular. Given the context of dental geriatric teaching within that particular module, the exposure to advanced clinical care in years 3 and 4 of the program and the focus of the preparedness model on this clinical care, first and second year students were not included in the study.

As the first author was the coordinator for the module who also graded the essays prior to this study, a staff member anonymized all the essays and only added an identifying code (and gender and class year). Although this was done to ensure anonymity, it also prevented any meaningful analysis in terms of age, race, ethnicity, urban/rural upbringing which would offer a richer interpretation of the findings. The same anonymized essays were then analyzed thematically for this study, using an inductive coding process to identify categories, subcategories, and themes of information until saturation was achieved as we have done previously [3, 6, 10, 11]. The evaluation of each essay for grading as part of a module had no relationship with the thematic evaluation that was conducted for this study.

We employed a qualitative inquiry method for the thematic analysis where anonymized essays were independently coded manually by the two authors (MB is a mid-career researcher and instructor for the undergraduate dental students; NR was a 2021 undergraduate summer student hired for this study). Although no qualitative software was used, we followed the coding guideline suggested by Saldana as an inductive process of identifying ideas in the form of a word or short phrase in the essays [12]. These words or short phrases—the codes—were then grouped into categories when expressing similarities; we proceeded to find relationships between these categories which together made up a main theme, as done in our previous studies [3, 6, 10, 13]. The first two essays were analyzed interactively by the two authors so that a coding scheme could be developed, and discrepancies on coding could be discussed until consensus was achieved; these discussions happened only via conference calls and electronic communications. We then proceeded independently with coding and the subsequent interpretation of patterns of meaning within the remaining essays. The two authors continued to occasionally meet virtually in case issues surfaced pertaining to coding, categories, and/or themes. Given the number of essays, only selected excerpts were used in the Results section to illustrate the themes. There was an attempt in choosing a variety of excerpts to represent views from both gender and academic years, in case there were differences between males and females and between seniority (4th year – graduating and 3rd year students).

## Results

The 111 essays ranged from 1499 to 2002 words each, generated more than 200 pages of text, and were analyzed thematically via an iterative coding process between June 10 and September 11, 2021. Given our objectives in encouraging students to consider the clarity, usefulness, comprehension, applicability, layout, and components of the model, the coding process led to the identification of four main themes that we judged fulfilled our goals: streamlined depiction, information-based approach, adaptability to an ever-changing situation, and room for improvement. Although the main themes do overlap in meaning, they are presented separately for easier conceptualization. In a qualitative study like this using a large number of somewhat lengthy essays, many other themes and codes would likely have surfaced but are not presented herein.

### Theme 1 – streamlined depiction

As prompted by the essay instructions, all students commented on its visual layout presentation. For 61 students, the arrangement and distribution of the text and arrows offered a “*streamlined visualization*” of what to expect, while in another essay, a third-year male student emphasized that “*for the model to act as a guide, it is important to be systematic so that everyone can easily follow the instructions step by step* [and] *accomplish this by presenting each main step or component and then consider what each of the components involves.*”

According to a fourth-year female student, the model “*visually reflects the position of the ‘context’ as a broad, encompassing factor that is the backdrop for all decision-making processes* [and] *the model contains itself to only a few examples of each type of context, which further reduces excess elements.*” For 103 students, the context of the pandemic was not just a thought, but something they vividly experienced in their personal and educational lives, as a fourth-year female student emphasised “*…the life-long learning and critical thinking aspects of the model are even more relevant to us during this pandemic, as we probably have more to learn after graduation given some of the training limitations we have experienced.*”

Furthermore, 89 students specifically commented on the usefulness of the arrows to show sequence and pace, one third-year student (male) further reflected that the “*constant back arrows remind me that this process is a cycle, rather than a single straight track from start to finish. This is important because a continuous refinement of the development process leads to a better final result… it is more streamlined and could function well as a constant reminder of what to take into account*”. As alluded to by at least 72 students, this continued development process concerning the ongoing pandemic hinges upon any knowledge and information one has or is exposed to, as presented in the next theme.

### Theme 2 – information-based approach

As an information-based model with various components, 68 students reflected on the lifelong learning characteristic of the profession as “*you are always, continuously evaluating the information in front of you, and the model emphasizes that such evaluation entails appraisal and summarization of material also during this pandemic*” (fourth-year female student). For others, including a third-year female student, “*information sets the tone to develop the awareness about the pandemic and its progress, infer the levels of risks you and your patients are at, and act accordingly for the benefit of all … now imagine when that information that started it all is still developing, evolving.*”

Concurrently, 23 other students pondered about the sorting process utilized during information processing. In particular, students seemed to be overwhelmed by the amount of facts and material available about the pandemic, especially when this information is contradictory. This was expressed by a third-year male student: “*the model shows source and type of information as relevant, but it has been overwhelming to read and listen to so much being said about the pandemic, and sometimes even different and conflicting views around the same topic. How* [can] *one best discern what is right or wrong?*” For another male student, from year 4, the issue was about the end result, as he wrote “*there are daily updates about the pandemic, and even more than once on the same day. This is too much. But isn’t the outcome the same, in terms of improving patient care and protecting them as we have been doing? How much more can we do when the information keeps changing?*”

The considerations brought up by students concerning the source, type, and quantity of information tests the ability of the providers to adapt to the ongoing changes surrounding the pandemic as highlighted by the theme below.

### Theme 3 – adaptability to an ever-changing situation

The evolving, ever-changing, and sometimes conflicting information about the pandemic itself and its impact on oral health care was considered by the students when thinking about the usefulness and applicability of the model. According to a fourth-year male student, “*the ongoing uncertainties about the pandemic would encourage a dentist to constantly refine and adapt, which is needed in a situation like this given the dynamics around it and rapid changes.*”

Indeed, the cascade of data and information about the COVID-19 prompted a third-year female student to state that “*the information that is constantly evolving about this pandemic requires us—dental providers—to engage in ongoing learning, much like a continuing education course or program so we keep the knowledge updated. The model should not be static because of that.*”

The potential change to the practice of dentistry, be it momentary or permanent, was also mentioned by 41 students when considering scheduling of patients, office layout, and personal protective equipment (PPE). More specifically, a fourth-year male student wrote: “*although optional before, face shields are here to stay I think, on the top of our surgical mask or respirators and loops to add safety to you and the patient.*” And on the particular issue of masks, when considering society-at-large, a third-year female student acknowledged the changes in public health orders: “*the use of masks for the general public can be seen under ‘risks’ if you do not wear one, but sometimes* [it] *can be confusing to follow what type of masks are better, and the restrictions that are in some indoor places, but not in others, and across different provinces … it is a constant change.*”

Overall, the idea of constant adaptation to a changing situation was conveyed in the words of 49 students when considering how the practice of dentistry has evolved over the years as part of the health care profession, and in the words of another 22 students when pondering re-evaluation in general: “*it is extremely important to reassess the whole situation as time passes, and more information is discovered and provided as depicted in the model*” (third-year male student).

Despite the positive views about the model, students recognized its limitations and suggested modifications and improvements presented ahead.

### Theme 4 – room for improvement

Students offered numerous ideas and suggestions to improve the model, from adding colour to the boxes and arrows (suggested by 14 students) to “*including additional dental-specific components that can appropriately improve our readiness to respond to a medical pandemic*” (fourth-year male student). For 11 students, the model was difficult to make sense of. In particular, one third-year male student wrote that “*…it still requires concerted effort to understand. Without any explanatory text, many of the assertions are presumptions and guesses on the model’s intentions,*” although the same student later emphasized that “*it still offers a useful guide on how to search out, evaluate, and gather information about a threat and then put that information into clinical action.*”

For another nine students, including a fourth-year female participant, the graphic portrayal presented could require some revision, as “*it is confusing whether the title headings are meant to display a range of characteristics, as its design seems to portray, or a more limited, fixed idea.*” In particular, the utilization of tele-dentistry in the model was acknowledged by 17 students, with 15 of them commenting on its financial aspects. More specifically for one third-year female student, consideration must be given to the patients “*who may see this technology as a simple virtual interview without expectation to be charged the same way as in a clinical appointment.*” For one fourth-year male student, “*with the emerging trend of tele-dentistry during the pandemic, there is a need to be more [clear] on the expectation of both parties and a guideline for its financial aspect, which can be incorporated into the model.*”

And four fourth-year students, three females and one male, took it a step further and provided their own models to exemplify their ideas. While two suggestions were very similar and offered more of a reorganization of the proposed model’s words and added illustrations, the two other students just described their models without an added portrayal. One student (male) ended his essay stating that “*oral health care should be the center of the model highlighting specifically a threat to it.*” And the fourth student, a female, justified her ideas by writing:

“*My model would work under the overarching idea that the pandemic leads to disruption, restricts access to our services by patients, and impacts our livelihoods. I would add the Public Health Agency of Canada ‘monitoring’ the pandemic as it serves the purpose of disseminating information and can be critical in the awareness. I strongly believe that these agencies have played a pivotal role during the pandemic in disseminating information and guidelines for healthcare workers as well as the general population, and they should be represented.*”

While students offered their own models, they also realized that the core message should remain the same as the initial model to foster preparedness via “*information and communication seeking behaviour, awareness and understanding of the pandemic, and the decision making to perform a particular oral health care strategy*” (fourth-year female student).

## Discussion

When asked “what is the applicability of a novel preparedness model for the provision of oral health care during an unfolding threat?”, senior undergraduate dental students at the University of British Columbia emphasized the model’s streamlined depiction, its focus on an information-based approach, and its adaptability to an ever-changing situation while still considering a need for improvement of the model. The engagement of undergraduate students is fundamental if we, as educators, aim to change practices that start at the beginning of a professional career. In fact, other models—or spatial representation of a concept—have been assessed by patients [11] students, [14] and professionals [6] although not with the intent to validate such models as a validity study [15]. As suggested by Kapborg and Fischbein, a model can “*work as a frame of reference* [and] *may function as a compass in an evaluation context when collecting, analysing and interpreting data as well as drawing conclusions*” [16]. We attempted to appraise this *compass* under the views of undergraduate dental students who would be soon practicing in the midst of an ongoing global health crisis. It is then essential to be prepared to deliver safe and informed oral health care beyond the current COVID-19 pandemic in the event of another outbreak.

It was encouraging to read that many students were aware of the ever-changing state of knowledge pertaining to the pandemic, which ultimately impacts preparedness as recognized by practicing dentists who suggested the initial model [6]. Preparedness can be understood as a set of actions taken in the face of disasters, from natural catastrophes to disease outbreaks [17], and has been widely discussed during the COVID-19 pandemic [18–20]. For the provision of oral health care in particular, preparedness can inform protocols [7] and models [6]. As discussed by Reddy and colleagues, “*protocols keep changing rapidly as new and more information of this novel disease is discovered*” [21]. Although such changes are due to the constant production of new evidence and data, some providers may find the amount of information about COVID-19 overwhelming and confusing at times [22], hindering the actual uptake of new knowledge [23]. Indeed the amount of sometimes unclear and contradictory information overload was discussed by the students when appraising the model and considering the COVID-19 crisis, and confirmed recently by Cox [24] and others [25].

As expected, many students reflected on the impact of the pandemic upon their education and training. There has been evidence highlighting the negative impact of the pandemic upon clinical dental training in particular, mainly due to limited and restricted access to patients and the switch of teaching approaches to distance learning [2–4, 26]. Students questioned their own preparedness to treat patients due to possibly limited clinical training, along with their preparedness to deal with the pandemic itself.

When commenting on the limitations and criticism of the model, numerous students contemplated tele-dentistry and its use. Although tele-health has been around for decades, and tele-dentistry has been referenced in the literature since the earlier 1990s [27, 28], many students raised concerns about its financial aspects which would likely influence its uptake. In fact, insufficient financial reimbursement has been cited as one of the main challenges related to its acceptance by the dental profession [29] (well before the COVID-19 pandemic). Nonetheless, the pandemic has placed tele-dentistry in the spotlight [30, 31] and oral health care providers seemed to be more aware of its value currently [32]. Unfortunately, students’ reasoning about the model seemed to still favour the somewhat siloed practice of dentistry as no student considered inter-professional education or interdisciplinary and collaborative work during this health crisis when all providers are working toward battling the pandemic and t improve the health care system.

Our study aimed at achieving rigour through reflexivity and data saturation, as we have employed elsewhere [3, 6]. Rigour ensured that the proposed research design, methods, and conclusions were clear and open to critique. We engaged in reflexivity by discussing the coding process and by describing the contextual relationships between the pandemic, the participants, and their education. Moreover, although saturation of information refers to the point during data collection where no new information was provided, we did collect and read all the 111 essays; as such, data did become repetitive even before coding all the essays in light of the study objectives.

Despite its strengths, this qualitative study is subjective and generalizability is unwarranted. Given that the essays were mandatory for course assessment purposes, the ethical standards of autonomy and voluntary withdrawal from the study could not be upheld. However, all identifiers (names, student number, etc) that could link the collected data to the respective students were removed to maintain confidentiality. Despite the relatively robust number of essays and data saturation, students were only from one dental school and do not represent all undergraduate dental students in Canada or elsewhere. In addition, the essays were produced for grading as part of a dental geriatric module and response bias may have occurred, that is, students might have given socially desirable answers they judged would be more acceptable by the instructor, rather than voicing what they really thought about the model which had been developed by that same module instructor. Nonetheless, the fact that students not only criticized the initial model but also offer their own models helps to minimize these biases. The four major themes presented are neither exhaustive nor representative of all viewpoints about the preparedness model and of the large number of codes produced; further analysis of the existing data, along with additional studies involving undergraduate dental students from other universities and practicing dentists are strongly suggested. The four alternative models suggested by students were not discussed in this manuscript, as they need to be critically appraised. Although we did not find any differences in the essays’ content based on gender or academic year of the students in this study, further analyses might unravel differences in ideas, understandings and pandemic considerations based on such characteristics.

Although preparedness to practice has been explored in health sciences [33, 34] and preparedness models have been evaluated by practicing dentists [6], to our knowledge this was the first time undergraduate dental students were engaged in appraising a preparedness model for the provision of oral health care during a pandemic. Students appeared to have a critical and enlightening view of preparedness models; as a result, however, at this time there is no teaching of pandemic preparedness per se in UBC undergraduate dental education.

## Conclusions

The current COVID-19 pandemic has significantly impacted oral health care services, and preparedness models can better guide oral health care providers during a health crisis. The recently developed preparedness model was appraised as useful by senior undergraduate dental students who soon will be practicing in the midst of a health crisis, and must be prepared. The thematic analysis revealed four major themes associated with the model. Although alternative portrayals were suggested, a comprehensive assessment of the newly developed model (and of its variations) by oral health care administrators/staff and patients is warranted to better support oral health care providers in delivering services during a pandemic.

## Data Availability

The de-identified data used and analysed during the current study are available from the corresponding author on reasonable request.

## Abbreviations

COVID-19: Coronavirus Disease 2019
DMD: Doctor of Dental Medicine
PPE: Personal protective equipment
SARS-CoV-2: Severe acute respiratory syndrome coronavirus 2
RISE: Researcher information services
UBC: The University of British Columbia
